# Influence of the Milling Strategy on the Marginal Fit of Chairside-Fabricated Lithium Disilicate Crowns

**DOI:** 10.3390/ma18102184

**Published:** 2025-05-09

**Authors:** Lara Berger, Felix Förtsch, Ralf Robert Kretschmer, Oleksandr Sednyev, José Ignacio Zorzin, Manfred Wichmann, Ragai Edward Matta

**Affiliations:** 1 Department of Prosthodontics, University Hospital Erlangen, Glückstrasse 11, 91054 Erlangen, Germany; lara.berger@uk-erlangen.de (L.B.); felix.foertsch@uk-erlangen.de (F.F.); ralf_kretschmer@t-online.de (R.R.K.); oleksandr.sednyev@uk-erlangen.de (O.S.); claudia.ehrhardt@uk-erlangen.de (M.W.); 2 Department of Operative Dentistry and Periodontology, University Hospital Erlangen, Glückstrasse 11, 91054 Erlangen, Germany; jose.zorzin@fau.de

**Keywords:** CAD/CAM, marginal fit, milling accuracy, lithium disilicate

## Abstract

Utilizing dental CAD/CAM systems, chairside treatments allow patients to be treated within one day without the need for an intermediate dental laboratory. With this procedure, it is also essential to ensure a sufficient marginal fit of the restoration. Therefore, this study investigates the influence of the milling strategy on the marginal fit of CAD/CAM-fabricated lithium disilicate crowns. For this purpose, 10 crowns were fabricated from each of the materials IPS e.max CAD and Celtra Duo using the fastest and finest available milling strategy. The accuracy of the marginal fit of the 40 crowns was examined using the industrial scanner ATOS Triple Scan and the associated software using the triple scan protocol. In a comparison of the milling processes, the restorations fabricated using the finest process always exhibited a better marginal fit, with mean deviations of 87 μm and 146 μm for IPS e.max CAD and 111 μm and 118 μm for Celtra Duo. The difference was only statistically significant for the crowns made of IPS e.max CAD (*p* = 0.008). All mean deviations determined were below the clinically acceptable marginal gap values (150 μm). Both materials can be used for chairside-fabricated crowns; however, choosing the faster milling strategy leads to higher marginal discrepancies.

## 1. Introduction

Progressive digitalization has revolutionized dentistry significantly since the 1980s, particularly through the introduction of dental CAD/CAM systems (Computer-Aided Design/Computer-Aided Manufacturing) [1]. These computer-aided technologies enable the precise and efficient processing of a wide range of materials, including ceramics, metals, composites, and plastics, with a particular focus on prosthetic applications [2]. In combination with intraoral scanners, these systems enable so-called chairside treatments, in which patients can be treated within one day without the involvement of a dental laboratory. Recent surveys show that in economically strong countries, more than a quarter of dentists already use this procedure, which points to its numerous advantages [3,4,5]. These include significant time and cost savings while maintaining or even improving the quality of the restoration, increased comfort for both patients and practitioners, and the overall increased efficiency of the entire workflow [6,7]. These developments set new standards in dental care and highlight the potential of digital technologies in modern dentistry [2]. The increasing use of chairside systems is closely linked to constant innovations in the field of dental ceramics, which have become indispensable as dental restorative materials due to their mechanical and esthetic properties and excellent biocompatibility. Dental ceramics can be divided into two main groups: oxide ceramics and glass ceramics. Oxide ceramics are characterized by very good strength properties but often involve esthetic compromises due to the opacities typical of the material. In contrast, glass-ceramics enable the fabrication of highly esthetic restorations, as they exhibit outstanding translucency with adequate mechanical properties, which is the main advantage of this material class [8].

Among glass-ceramics, lithium disilicate-based materials in particular have established themselves, achieving a flexural strength of 350 MPa to 450 MPa and thus exhibiting significantly increased mechanical properties compared to leucite-reinforced ceramics (80 to 150 MPa) or feldspar ceramics (100 to 160 MPa) [8,9]. The structural properties of this material class have considerably expanded the range of indications for glass-ceramics, so that monolithic lithium disilicate blocks are now not only used for single-tooth crowns, but can also be used for the fabrication of fixed partial dentures (FPDs) in the anterior region and for three-unit FPDs with premolar involvement [10,11,12]. However, the strength is not yet sufficient for longer-span restorations in the posterior region, which is why the optimization of the mechanical properties of glass-ceramics is a focus of current research [10,13,14]. A key approach to increasing the strength characteristics of glass-ceramics is to further improve crack resistance, as crack propagation is one of the main causes of failure in glass-ceramic restorations. This is where the class of zirconia-reinforced lithium disilicate ceramics comes in, as zirconia undergoes a volume increase of 3–5% when subjected to extreme forces due to a phase transformation from the monoclinic to the tetragonal structure. This increase in volume generates a compression pressure that can inhibit crack propagation and thus contribute significantly to the stability of the ceramic [13]. Recent reviews have shown that these materials are a good alternative to conventional lithium disilicate ceramics. When comparing the mechanical properties of the two material classes, however, the data situation is ambiguous, so further research is still required to classify the mechanical properties more precisely [15,16].

In addition to the material properties, various clinical quality characteristics have a direct influence on the long-term stability of dental restorations, including the accuracy of fit of the crown on the tooth [17]. A seamless transition between the crown and tooth would be ideal, but this cannot be achieved in practice due to technical limitations in the manufacturing process and the need for a spacer for the luting material between the tooth and crown. This inevitably results in a narrow gap, which is referred to as the marginal gap. This should be kept as small as possible, as too large a discrepancy promotes the accumulation of plaque and colonization by cariogenic microorganisms, which in turn increases the risk of secondary caries [18,19]. In addition, over-contoured or protruding crown margins can irritate the gingiva and lead to irreversible periodontal damage in the long term [20,21]. In the scientific literature, the information on a clinically acceptable range of the marginal gap value varies, with the upper limit typically being between 100 μm and 120 μm [22,23,24]. Marginal gaps of less than 120 μm can be realized realistically with common CAD/CAM systems [25,26].

In addition to conventional two-dimensional methods such as the silicone replica technique or the cross-section method, various digital three-dimensional methods are now available for measuring the marginal gap, which enable an even more precise and comprehensive examination of the accuracy of fit. In addition to optical coherence tomography and micro-computed tomography, these methods also include the triple scan method described by Holst, Matta et al., which was used in this study [27,28].

Another decisive factor for the longevity and mechanical stability of ceramic restorations is the surface quality. Industrially manufactured monolithic ceramic blocks, which can be processed using the CAD/CAM technique, are characterized by a high degree of material homogeneity [29]. Nevertheless, the milling process during fabrication can create fine microcracks both on the surface and inside the material, which can act as potential fracture initiation points and compromise the mechanical integrity of the restoration [30,31]. Fractographic analysis of clinically failed all-ceramic restorations has shown that such defects are often the starting points for restoration failures [32,33].

The quality of the milling process is influenced by various factors, including the size, shape, and abrasiveness of the burs, the number of axes of the milling machine, the production mode, and the selected milling strategy [31,34,35]. A recent review has shown that five-axis milling machines deliver more precise results in terms of accuracy of fit than machines with three or four axes. In addition, the wear of the burs, regardless of their geometry, affects the accuracy of the manufacturing process [34]. The milling strategy also plays a decisive role: depending on the material used and the manufacturer’s recommendations, at least one fast and one fine milling strategy should be implemented. While fast milling strategies are associated with a time saving of around ten minutes, the question arises as to whether this reduces the precision of the restoration [34,36].

The aim of the present in vitro study was, therefore, to investigate the influence of different milling strategies on the marginal fit of CAD/CAM-fabricated monolithic lithium disilicate crowns made of IPS e.max CAD and Celtra Duo. The null hypotheses were that neither the milling strategy nor the selected restorative material had an influence on the accuracy of fit.

## 2. Materials and Methods

To conduct this study, 40 composite tooth dies (Grandio Disc, VOCO GmbH, Cuxhaven, Germany) were fabricated, which were based on an ideally prepared maxillary canine with a chamfer preparation. To subsequently design and fabricate identical crowns, the software of the CEREC system (Dentsply Sirona, Charlotte, NC, USA) required information about the positions of the tooth die and the adjacent teeth. For this reason, a scan model was created into which the fabricated tooth dies could be reproducibly integrated and removed for digital impression taking. The dies and the corresponding scan model were matted with titan oxide spray (CEREC Optispray, Dentsply Sirona) to enable precise and fast digital capture [37] and then digitized with the CEREC Omnicam (Dentsply Sirona) by an experienced clinician.

The greatest possible equivalence of the crowns was crucial for the comparability of the individual crowns when examining the accuracy of fit. To ensure this, the crown shape was calculated using the factory settings (Table 1) stored by the manufacturer for the respective material in the CEREC SW 4.4.0 software (Dentsply Sirona) and the “biogeneric” function. It was checked whether the minimum thickness was met at all points before proceeding to crown fabrication. The materials IPS e.max CAD LT A2 C14 (Ivoclar Vivadent, Schaan, Liechtenstein) and Celtra Duo LT A2 C14 (Dentsply Sirona) were each processed with the CEREC MC XL (Dentsply Sirona) CNC unit for the fabrication of 20 crowns. For both materials, 10 crowns were fabricated using the fastest and finest milling strategy available: for the IPS e.max CAD material (Ivoclar Vivadent), the milling strategies were fast (Group A) and extra-fine (Group B), and for Celtra Duo (Dentsply Sirona), fine (Group C) and extra-fine (Group D). The mean manufacturing times for each group are listed in Table 2. For the crowns made of IPS e.max CAD (Ivoclar Vivadent), a crystallization firing was conducted at 850 °C for 30 min in the CEREC Speedfire (Dentsply Sirona) ceramic furnace in accordance with the manufacturer’s instructions to achieve the final hardness of the material required for insertion. According to the manufacturer, this step was not necessary for the crowns made of Celtra Duo (Dentsply Sirona).

The total of 40 crowns and 40 dies were digitized for the following analyses, both individually and in combination with the high-resolution, light-optical ATOS Triple Scan (Carl Zeiss GOM Metrology GmbH, Braunschweig, Germany), whose average measurement error during object registration is only 3 μm [28]. According to the scan protocol from the pilot study by Holst, Matta et al. [28], each scan series consisted of three individual scans: the crown alone, the die alone, and the respective crown on the corresponding die. For the scan of the crown on the corresponding die, the crown was reproducibly fixed with constant pressure in a special device using a pin in the central part of the crown. The captured data were transferred directly to a connected computer in the ATOS Professional 2016 software (Carl Zeiss GOM Metrology GmbH) to be able to combine the individual scans into one object and subsequently perform the matching of the scans and the analytical evaluation.

The marginal fit was analyzed both three-dimensionally using a marginal surface analysis and two-dimensionally in the form of a virtual cross-sectional analysis. For the three-dimensional marginal surface analysis, an area between the preparation margin and an offset curve located 1 cm further into the lumen was defined. This area was compared for each die with the surface of the corresponding inside of the crown, and the fit was calculated based on distance measurements. The mean deviation of the marginal measuring area was determined as the arithmetic mean of all measured orthogonal distances from the die surface to the inside of the crown (“Mean” in μm).

**Figure 1:**
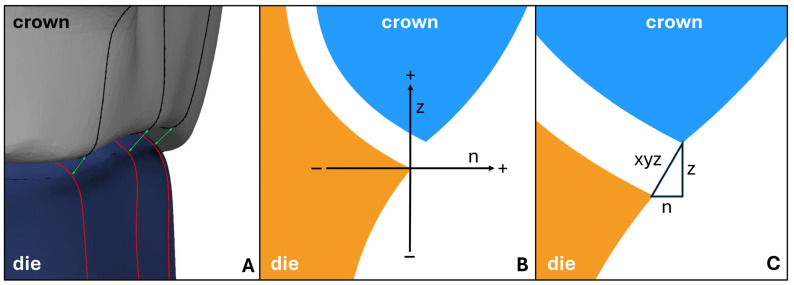
Two-dimensional cross-sectional analysis: (**A**) Visualization of the sections (red/black) and the absolute marginal discrepancy (green). (**B**) Illustration of the vectors of the horizontal (n) and vertical (z) marginal discrepancy. (**C**) Visualization of the measurement distances after Holmes [38].

Within the two-dimensional cross-sectional analysis, which was also carried out using the software ATOS Professional (Carl Zeiss GOM Metrology GmbH), the linear discrepancies between the crown margin and the preparation margin were recorded with the help of pre-programmed analysis scripts. The program generated 20 radial sections at 18° intervals, starting from the center of the crown or die. For each section, three distances were calculated according to the classification by Holmes et al. [38]: the “z” distance along the vertical z-axis of the coordinate system (vertical marginal discrepancy), the “n” distance in the horizontal plane (horizontal marginal discrepancy), and the “xyz” distance as the absolute linear distance between the crown margin and the preparation margin (absolute marginal discrepancy), as illustrated in Figure 1.

**Figure 2:**
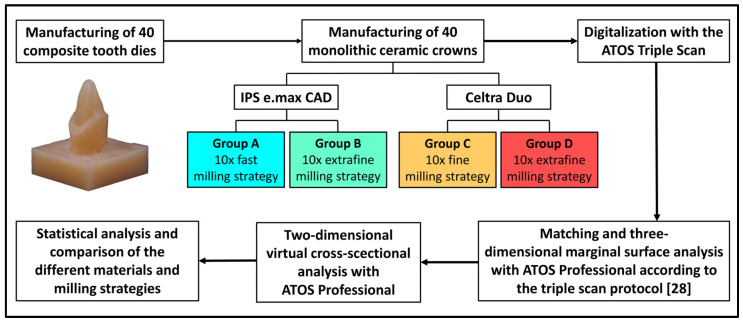
Overview of the study design.

A Wilcoxon signed rank test was performed for the statistical analysis of the acquired data using the statistical software SPSS (version 28.0.3, IBM Corporation, Armonk, NY, USA), with the significance level being set at 0.05. A graphical overview of the study design can be found in Figure 2.

## 3. Results

### 3.1. Three-Dimensional Marginal Surface Analysis

**Figure 3:**
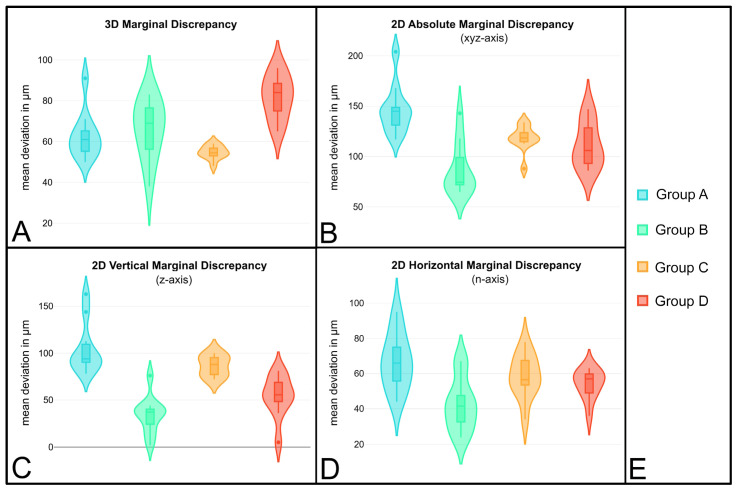
Violin plots showing the mean distance in μm for all groups (**E**) for the three-dimensional marginal discrepancy (**A**) and the two-dimensional absolute marginal discrepancy (**B**), as well as the vertical (**C**) and horizontal (**D**) marginal discrepancies.

In the marginal surface analysis, smaller discrepancies were found for both materials when selecting the faster milling strategy (Table 3, Figure 3). However, these results were only statistically significant for the crowns made of Celtra Duo (Dentsply Sirona) (Table 4). In the material comparison, the extra-fine milled crowns made of IPS e.max CAD showed a significantly higher precision with a 16 μm lower mean deviation.

### 3.2. Two-Dimensional Virtual Cross-Sectional Analysis

In the two-dimensional virtual cross-sectional analysis, the deviations in relation to the vertical (z-axis), horizontal (n-axis), and absolute (xyz-axis) marginal discrepancy were examined and compared. For both materials, the restorations fabricated using the fastest possible milling strategy showed a higher deviation in all three axes (Table 5, Figure 3).

When the absolute marginal discrepancy was examined, only the comparison of the crowns made of IPS e.max CAD with a difference in the mean deviations of 59 μm was statistically significant (Table 6); for the crowns made of Celtra Duo (Dentsply Sirona), the difference was only 7 μm. In the material comparison, the restorations made of IPS e.max CAD (Ivoclar Vivadent) showed significantly higher accuracy, with the mean deviation in the xyz axis being 24 μm lower.

The same results were obtained for the vertical and horizontal marginal discrepancy. All comparisons were statistically significant in the z-axis, but only the comparison of the milling strategies for crowns made of IPS e.max CAD (Ivoclar Vivadent) was significant in the n-axis.

## 4. Discussion

In the scientific examination of the accuracy of fit of crowns, there are various methodological approaches that are characterized by individual influencing factors that significantly determine the accuracy and clinical relevance of the analyses [39,40,41,42]. A key aspect is the distinction between in vivo patient studies and in vitro laboratory studies. In in vivo studies, the fabrication of crowns requires customized tooth preparations that vary from patient to patient, potentially limiting the comparability of data. In addition, patient-specific challenges during impression-taking can affect the accuracy of measurements [43]. In contrast, in vitro analyses allow the fabrication of impressions and the corresponding crowns under standardized conditions, which simplifies a differentiated evaluation of the accuracy of fit. This is also reflected in the fact that a large proportion of the investigations into the accuracy of fit of crowns are conducted as in vitro studies [44].

The selected restorative material and the manufacturing process also affect the accuracy of fit. Nowadays, the dental market offers a wide range of products for monolithic glass-ceramic restorations manufactured using the CAD/CAM workflow, so it is necessary to conduct studies to verify the material properties stated by the manufacturer and thus to find the best possible materials for the clinical application [8,45]. Since both IPS e.max CAD (Ivoclar Vivadent) and Celtra Duo (Dentsply Sirona) are frequently used in chairside procedures in everyday clinical practice [46], these materials were to be investigated in the present study.

The choice of measurement method is a crucial factor in the significance of the study, whereby Nawafleh et al. recommend a combined measurement method for analyzing the marginal fit in a review [44]. The triple-scan protocol introduced by Holst, Matta et al. [28] enables the generation of a large number of measurements per crown during three-dimensional surface analysis, which significantly increases the informative value of the fit analysis. In addition, the data generated by the scanning protocol can be used to perform a further virtual two-dimensional cross-sectional analysis, which eliminates potential sources of error in the production of replicas and sections. Both measurement methods complement each other; critical areas with unacceptable margins are more easily identified in the two-dimensional analysis, while the three-dimensional surface analysis, due to the high number of measured values, may hide potentially unacceptable deviations within acceptable average values. Conversely, large deviations in the individual measurements of the two-dimensional analysis can make a precise assessment of the fit more difficult. Accordingly, it can be assumed that the combined measurement methodology can ensure a sufficiently high level of validity for the study [40,47,48].

The first null hypothesis was that the choice of milling strategy has no influence on the marginal fit of the restoration. This hypothesis must be rejected since the crowns milled in the fastest possible milling process showed higher marginal discrepancies in the two-dimensional cross-sectional analysis. When the absolute marginal discrepancy (xyz-axis) was considered, all mean deviations determined were below the clinically acceptable value of 120 μm [22,24], except for the fast-milled crowns made of IPS e.max CAD. Other authors consider margins of up to 150 µm to be sufficient [49]. However, the maximum deviation in group A significantly exceeded this value at 204 µm. For all other groups, the maximum deviation remained below 150 µm. The differences between the various milling strategies were statistically significant only for the crowns made of IPS e.max CAD, with a difference in the mean deviation of 59 μm (*p* = 0.008); for the crowns made of Celtra Duo (Dentsply Sirona), the difference was only 7 μm (*p* = 0.26).

The results of this study, with a mean deviation of 87 and 146 μm for IPS e.max CAD (Ivoclar Vivadent) and 111 and 118 μm for Celtra Duo (Dentsply Sirona), are in the same range as the results of similar comparative studies [39,49,50,51,52,53]. For IPS e.max CAD (Ivoclar Vivadent), values between 55 μm [39] and 146 μm [53] are found in the literature, while for Celtra Duo (Dentsply Sirona), the range includes values from 46 μm [52] to 131 μm [49]. These considerable differences can be attributed to different manufacturing parameters in crown production and differences in the measurement methodology. However, the chosen manufacturing strategy was not mentioned in any of the comparative studies, making it difficult to critically evaluate the differences identified in this work.

When the individual axes were examined, the crowns manufactured using the extra-fine grinding process always showed less deviation, although the comparison in the horizontal axis for the crowns made of Celtra Duo (Dentsply Sirona) was not significant (*p* = 0.332). There is also hardly any comparative data available here; some results for IPS e.max CAD (Ivoclar Vivadent) can be found for the vertical discrepancy alone. In a study by Neves et al., this was 39 ± 9 μm [54]; Mostafa et al. found a value of 33 ± 20 μm in their study [55]. Both results are within the range of values in this study of 19 ± 37 μm and 105 ± 28 μm, respectively. However, no information on the milling strategy used can be found in the studies, either.

When comparing the milling strategies as part of the three-dimensional marginal surface analysis, the crowns produced in the respective fastest possible process proved to be more precise, whereby the difference with a mean deviation of 27 μm was only significant for the restorations made of Celtra Duo (Dentsply Sirona) (*p* = 0.005). For the crowns made of IPS e.max CAD (Ivoclar Vivadent), the results were very close to each other, with deviations of 63 ± 11 μm and 66 ± 14 μm, respectively (*p* = 0.374). This three-dimensional marginal surface analysis considers a 1 cm wide area starting from the preparation margin, which is referred to as the “internal marginal gap” according to Holmes et al. [38]. Within this area, over 10.000 individual measurement sections are defined, from which the mean value is calculated. In contrast, the two-dimensional virtual cross-sectional analysis evaluates the absolute marginal discrepancy, i.e., the actual distance between the cervical restoration margin and the preparation margin. In addition, the vertical and horizontal marginal discrepancies can be viewed in isolation by vector determination. Due to these facts, this two-dimensional analysis method can be considered more relevant with regard to the marginal fit of the restoration.

The second null hypothesis was that the selected restorative material has no influence on the marginal fit. This hypothesis must also be rejected since the restorations made of Celtra Duo (Dentsply Sirona) showed significantly higher marginal discrepancies in both analysis methods. In the three-dimensional analysis, the difference in mean deviations was 16 μm (*p* = 0.015), and in the two-dimensional analysis, it was 24 μm (*p* = 0.022) for the absolute marginal discrepancy. In a recent study, Elsayed et al. also found a higher marginal fit for IPS e.max CAD (Ivoclar Vivadent) compared to Celtra Duo (Dentsply Sirona) [39], but studies with opposite results can also be found [51,52]. This inconsistent data situation can be attributed to different study designs and the associated challenges in the comparability of the measurement methods. It is also possible that these differences are influenced by other factors, such as the thickness of the titanium oxide powder layer or the positioning of the crowns on the dies [56].

Despite the informative results of this study, its limitations must also be considered. For one thing, the selected crown shape could have influenced the milling precision since the milling machine may have had more difficulty reaching some areas depending on the geometry of the restoration. For another, although the two materials examined are frequently used, they represent only a small part of the product range of dental glass-ceramics [10].

Regarding future research approaches, the investigation of CAD/CAM-fabricated glass-ceramic crowns in relation to the possible influence of the crown material and the manufacturing strategy could be extended by considering additional parameters in addition to the accuracy of fit. These include surface quality, wear behavior, and survival rates, which could be determined using chewing simulators. Such an extension of the analysis would enable well-founded conclusions to be drawn that would allow a differentiated recommendation for clinical application. Given the increasing use of monolithic single-tooth crowns made of different composite or zirconia blocks in clinical practice, it would also be useful to examine these materials in more detail in the context of the present work. This could provide valuable insights for practical application and help to optimize treatment outcomes.

## 5. Conclusions

Considering the limitations of this in vitro study, the following conclusions can be drawn:

▪ Choosing the faster milling strategy leads to higher marginal discrepancies, regardless of the material used
▪ When fabricating crowns from IPS e.max CAD (Ivoclar Vivadent), choosing the fast milling strategy can result in clinically unacceptable marginal gap values
▪ The smallest marginal discrepancies were achieved with the material IPS e.max CAD when the extra-fine milling strategy was selected
▪ Both materials investigated are suitable for crown fabrication using the chairside technique if the appropriate milling strategy is applied

When using lithium disilicate ceramics chairside, it is important to consider whether the time saved by choosing the faster milling strategy justifies the risk of clinically unacceptable marginal gap values.

## Figures and Tables

**Table 1:** Factory settings in the CEREC software for IPS e.max CAD (Ivoclar Vivadent) and Celtra Duo (Dentsply Sirona).

Parameter	Default Value
Spacer (radial)	120 μm
Spacer (occlusal)	120 μm
Proximal Contact Strength	25 μm
Occlusal Contact Strength	25 μm
Dynamic Contact Strength	25 μm
Margin Ramp Angle	60°
Minimal Thickness (radial)	1000 μm
Minimal Thickness (occlusal)	1500 μm
Margin Thickness	50 μm

**Table 2:** Mean manufacturing times for all groups.

Material	Milling Strategy	Mean Manufacturing Time	Group
IPS e.max CAD	fast	8 min	A
	extrafine	22 min	B
Celtra Duo	fine	14 min	C
	extrafine	22 min	D

**Table 3:** Descriptive statistics of the three-dimensional marginal fit analysis: mean deviation (Mean) with standard deviation (SD) and the minimum (Min) and maximum (Max) deviation in μm for all groups.

Group	Mean [μm]	SD [μm]	Min [μm]	Max [μm]
A	63	11.2	50	91
B	66	14.2	38	83
C	55	3.3	48	59
D	82	10.6	65	96

**Table 4:** *p* values for all statistical comparisons as part of the three-dimensional marginal fit analysis.

Variable	Group 1	Group 2	*p* Value
Milling strategy	A	B	0.374
	C	D	0.005
Material	B	D	0.015

**Table 5:** Descriptive statistics of the two-dimensional virtual cross-sectional analysis: mean deviation (Mean) with standard deviation (SD) and the minimum (Min) and maximum (Max) deviation in μm for all groups.

Group	Axis	Mean [μm]	SD [μm]	Min [μm]	Max [μm]
A	xyz	146	24.8	117	204
	z	105	27.9	78	163
	n	67	16	44	95
B	xyz	87	26	65	143
	z	19	36.5	−76	44
	n	43	15	24	67
C	xyz	118	12.8	88	134
	z	86	10.8	72	100
	n	59	12	34	78
D	xyz	111	21.9	86	147
	z	54	22	5	81
	n	54	8	36	63

**Table 6:** *p* values for all statistical comparisons as part of the two-dimensional virtual cross-sectional analysis.

Axis	Variable	Group 1	Group 2	*p* Value
xyz	Milling strategy	A	B	0.008
		C	D	0.26
	Material	B	D	0.022
z	Milling strategy	A	B	0.007
		C	D	0.005
	Material	B	D	0.005
n	Milling strategy	A	B	0.022
		C	D	0.332
	Material	B	D	0.066

## Data Availability

The underlying data are available from the corresponding author upon reasonable request.
